# Tumor Restrictions to Oncolytic Virus

**DOI:** 10.3390/biomedicines2020163

**Published:** 2014-04-17

**Authors:** Markus Vähä-Koskela, Ari Hinkkanen

**Affiliations:** 1 Institute of Biotechnology, University of Helsinki, Helsinki 00790, Finland; 2 A.I. Virtanen Institute for Molecular Sciences, University of Eastern Finland, Kuopio 70211, Finland

**Keywords:** oncolytic virus, interferon, tumor stroma, antiviral defenses, extracellular matrix, tight junctions

## Abstract

Oncolytic virotherapy has advanced since the days of its conception but therapeutic efficacy in the clinics does not seem to reach the same level as in animal models. One reason is premature oncolytic virus clearance in humans, which is a reasonable assumption considering the immune-stimulating nature of the oncolytic agents. However, several studies are beginning to reveal layers of restriction to oncolytic virotherapy that are present before an adaptive neutralizing immune response. Some of these barriers are present constitutively halting infection before it even begins, whereas others are raised by minute cues triggered by virus infection. Indeed, we and others have noticed that delivering viruses to tumors may not be the biggest obstacle to successful therapy, but instead the physical make-up of the tumor and its capacity to mount antiviral defenses seem to be the most important efficacy determinants. In this review, we summarize the constitutive and innate barriers to oncolytic virotherapy and discuss strategies to overcome them.

## 1. Introduction

In most solid tumors the tumor interior displays a high cellular density with multiple cell types, both malignant and normal (stromal) cells, interdigitated by strands of extracellular matrix (ECM) and other poorly defined tissue. Stromal cells typically consist of fibroblasts, macrophages, neutrophils and other immune cell types. Tumor-associated fibroblasts are a source of the bulk acellular tissue in tumors [1], which is largely made up of polysugars and glycoproteins, such as collagens, laminins, fibronectins, proteoglycans, hyaluronan and water, forming a dense matrix around and between the cancer cells [2]. This matrix provides structural support but also haptotactic and chemotactic guidance for cancer cells [3]. Moreover, the prevailing tumor interstitium is rich in soluble angiogenic factors, such as members of the vascular endothelial growth factor (*VEGF*) family [4], cellular growth factors, including endothelial growth factor (*EGF*), basic fibroblast growth factor (*FGF*), platelet-derived growth factor (*PDGF*) and pleiotrophic factors, particularly transforming growth factor (*TGF*) beta [5]. Additionally, both tumor- and stromal cells secrete cytokines and chemokines, such as interleukin (IL) 6, IL 10, tumor necrosis factor (*TNF*) alpha, stromal cell-derived factor (*SDF*) 1, macrophage migration inhibitory factor (*MIF*) 1, which on one hand recruit more stromal and progenitor cells into the tumors and on the other hand curb the activity of antigen-presenting cells and anti-tumor T cells [6,7,8]. In several independent cancer types, high serum levels of IL 6 and IL 10 are indicators of poor prognosis [9].

Aberrant tumor and stromal growth combined with an overproduction of matrix leads to high interstitial pressure in the tumor, preventing lateral diffusion of therapeutic compounds within the tumor. Tight cellular compactness combined with a high tumor proliferation rate also causes physical crowding and puts constraints on oxygenation, which due to aberrant angiogenesis and rupture of the haphazard vessels is mostly incomplete and results in chronic tissue hypoxia toward the tumor cores. As cancer cells exist in a crowding/hypoxic- and cytokine/growth factor-perpetuated chronic state of stress, they upregulate a number of key cellular molecules that function as a collective defense against a wide range of therapies, including oncolytic viruses. The properties of oncolytic viruses and their status in clinical development as well as various delivery options have been reviewed elsewhere [10,11,12,13]. In this review, we provide an overview of two of the main intratumoral barriers to oncolytic virus spread; the extracellular tumor make-up and the intracellular antiviral defense mechanisms.

## 2. Physical Barriers to Oncolytic Viruses

In this chapter we introduce the principal physical obstacles for successful oncolytic virotherapy and discuss ways to overcome them. As viruses lack autonomous motility and may potentially adsorb to any surfaces that display their specific receptors, they may only infect the physically delimited regions where they first entered and they may be prone to unfruitful sequestration by already dead cells. Nevertheless, oncolytic viruses vary in size and some are small enough to fit even through tight ECM networks, and efforts have been made through genetic engineering of the viruses to minimize their non-specific binding properties. Most interestingly, viruses may be engineered to express enzymes that directly break down the physical barriers to oncolytic viruses.

### 2.1. Interstitial Fluid Pressure

Tumor cellular structure and composition may in concert with abundant extracellular matrix (ECM) deposition pose a severe hurdle for systemic virus entry and propagation of infection within the tumor. The extracellular matrix is a complex dense network consisting of multiple proteins, glycoproteins and polysaccharides including collagens, laminins, fibronectins, proteoglycans and hyaluronic acid [14]. The interstitial fluid pressure (IFP) in the tumor surrounding blood and lymphatic vessels is mainly created by the high cell density that forms an increased physical pressure outwards and does not allow free diffusion of therapeutics [15,16,17]. A high IFP generally predicts an aggressive tumor phenotype as tumor cells tend to escape from tumor margins where the pressure drops, thus facilitating spread of metastatic cells [18]. On the other hand, for therapeutic virus, interstitial fluid concentration gradients may pose a hurdle as passive diffusion in viable regions, typically the rim, may occur mainly outward [19]. Given the limited arsenal of known chemicals alleviating tumor compactness and the poor penetration drugs in general into the tumor tissue, this is a considerable problem that may not be easy to tackle. Some strategies are discussed below.

### 2.2. Extracellular Matrix Deposits

**Figure 1:**
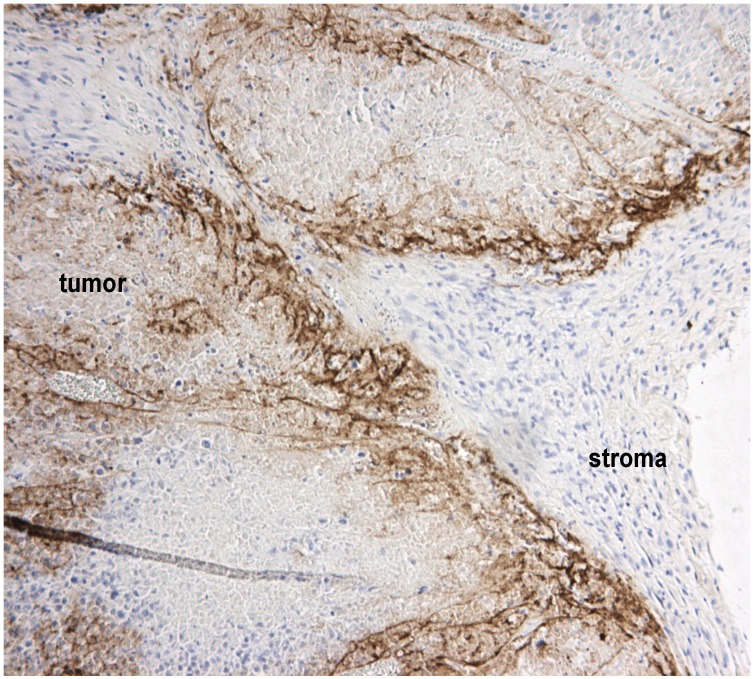
Tumor stroma blocks virus spread within tumors. A representative section of human A2058 melanoma xenografts stained with polyclonal Semliki Forest virus (SFV) antibodies (in brown) shows that even following intratumoral injection, virus infection is delimited by non-permissive stromal cells and the extracellular matrix (collectively called stroma). We have studied these barriers in detail previously [25].

Viruses are passive particles and rely either on radial cell-to-cell spread or on soluble diffusion across concentration gradients to reach their target cells and to propagate the infection. Tumor matrix is critical in this regard, as it can block both forms of spread. Passively diffusing viruses may physically not fit through the strands of the ECM. This was shown for oncolytic herpes virus, which has an outer diameter of over 100 nm, and whose spreading could be improved by matrix-degrading bacterial collagenase co-injection [20]. Bilbao and coworkers showed that adenovirus entry into experimental hepatocellular carcinoma nodules in the livers of immunocompetent rats following intravenous or intraportal injection was directly related to the thickness of the ECM capsule enveloping the nodules [21]. On the other hand, we found using Semliki Forest virus, which has an outer diameter of about 60 nm, that even upon intratumoral injection spread was blocked by ECM, which formed a physical barrier for virus infection (Figure 1).

The foremost option to reduce interstitial fluid pressure and to remove physical molecule barriers imposed by the ECM is to use matrix-degrading enzymes. These act by digesting collagen-, fibrin- and other types of fibrillar matrix deposits, creating more space between cell clusters, and glycosaminoglycan polymers, known to limit fluid movement in tumors. Simultaneously, such strategies may also expose more cell surface to viruses, increasing the likelihood of infection. In a study by Kuriyama, trypsin or collagenase/dispase was injected into subcutaneous U87 and U251 glioma xenografts in immunocompromised mice, followed by a reporter adenovirus [22]. Both types of ECM-degrading enzymes increased tumor transduction by the virus, but when doses got too high, transduction efficacy suffered, demonstrating a balance between ECM-degradation and oncolytic virus efficacy. In another study, treatment of PC3 tumors with vaccinia virus producing matrix metalloprotease (*MMP*) 9 resulted in reduction of collagen IV fibrils and increase in virus penetration into tumor, which yielded elevated virus titers [23]. Hong *et al*. targeted orthotopic neuroblastoma xenografts engineered to express *MMP9* with oncolytic HSV, achieving increased virus distribution compared to control tumors [24]. Similar results were obtained when human soft tissue sarcoma HSTS26T overexpressing *MMP1* or *MMP8* were injected with oncolytic HSV [19]. MMP1/8-expressing tumors contained significantly less sulfated glycosaminoglycans compared to control tumors. 

Other enzymes and protein effectors, either engineered into virus expression cassettes or provided exogenously, include hyaluronidase, decorin, various other MMPs, and notably relaxin—a peptide hormone normally expressed during particular phase of pregnancy that does not possess tissue-degrading activity itself, but instead induces a number of key collagen-degrading proteases seemingly in a tumor-specific manner [26]. Beyer showed that relaxin expressed by murine stem cells improved trastuzumab penetrance and therapy outcome in syngeneic tumor models [27]. In another study, chimeric adenovirus Ad5/35 expressing relaxin showed increased tumor transduction and virus dissemination [28].

Several other strategies to increase penetration of therapeutics in tumors have been developed [29]. While the stromal/ECM capsule of tumor nodules acts as a physical barrier to virus entry following intravenous injection, it was possible to improve tumor infection by administering vaso-active compounds (angiotensin II, histamine, nitroglycerine) before the virus [21]. This is interesting, as vasculature per se would not be expected to alter or influence the physical composition of the ECM, such as collagen strand thickness. Instead, virus access to the tumor was improved, most likely by increased access from the blood via tumor-stromal-adjoining vessels. Interestingly, in a study with oncolytic herpes virus, anti-VEGF monoclonal antibody Bevacizumab given before intravenous virus injection gave poorer anti-tumor efficacy than when given after the virus [30]. The study showed that while HSV is able to enter and infect tumors better through their leaky vasculature compared to Bevacizumab-normalized vasculature, vascular normalization by Bevacizumab still gave superior combination efficacy when the virus was already in the tumor. This effect was likely dependent on altering both physical and biological properties of the tumor, including interstitial pressure and oxygenation. In another study, anti-VEGF-A antibody injections in nude mice harboring U251 human glioma xenografts led to an increase in *MMP2* expression and reduction in collagen fiber content, facilitating improved distribution of oncolytic adenovirus within the tumor tissue [31]. In general, targeting tumor vasculature by oncolytic viruses and including agents that affect tumor vasculature in combination regimens is gaining interest [32], and it will be interesting to see more specific studies on how vascular-acting agents may affect the physical barriers to oncolytic viruses.

Some viruses do not need an extracellular step for propagating infection, such as members of the herpes and poxvirus families, and may be able to spread from cell-to-cell. For example, HSV-1 is able to infect neighboring cells via lateral tight junctions in a manner dependent on its glycoproteins E and I [33]. Vaccinia virus, on the other hand, induces so called actin-tails, which are actin-filament-driven membranous protrusions of the plasma membrane harboring a single virus particle at the outer tip. These actin tails actively deliver and deposit virus particles onto or even into neighboring cells [34,35]. Because lateral spread without an extracellular step may circumvent neutralizing immunity as well as some of the physical obstacles the tumor microenvironment imposes, this ability has been engineered into viruses that normally do not possess it. A promising strategy is to engineer OVs to express membrane-fusogenic genes (MFGs), such as gibbon ape leukemia virus (GALV) glycoprotein, reptilian reovirus p14 protein (*FAST*) or the membrane glycoproteins H and F of measles virus [36,37]. Quite intriguingly, expression of several different MFGs by oncolytic adenovirus synergized with chemotherapy in anti-tumor efficacy both *in vitro* and *in vivo* [38], suggesting that membrane fusion facilitated lateral spread of also chemotherapeutics, which otherwise would not have occurred in a compact tumor. The potential limitation of cell-to-cell-dependent spread, however, may be that membrane-free physical barriers, such as the ECM, may still pose a barrier to spread. Also, it is unclear whether MFGs or other mechanisms of lateral spread may assist in reaching distant tumor nests.

Some other strategies to alleviate matrix-imposed restrictions to oncolytic viruses have been discussed elsewhere [26,29].

### 2.3. Tight Junctions Block Virus Penetrance and Hide Virus Receptors

Tumors of epithelial origin mostly retain the firm cellular integrity seen in their original adhesive intercellular configuration. Several viruses use cellular receptors which are located in paracellular tight junctions, which may be problematic in tightly packed tumors [11]. Adenovirus C group viruses use coxsackie-adenovirus receptor (*CAR*) as their primary entry point, whereas adenovirus B1 group members and measles virus Edmonston use CD46 complement binding molecule as their cellular receptor [39]. Adenovirus B2 group (serotypes 3, 7, 11 and 14) entry occurs using desmoglein-2 (*DSG-2*) [40]. Of these, *CAR* and *DSG-2* are preferentially located within tight junctions and are hidden from virus binding [41]. Also the complement receptor CD46 was ascribed a role in maintenance of epithelial cell integrity by interactions with the E-cadherin/catenin network [42]. These observations imply that before reaching the potential entry site on the tumor cell the virus must find its way into the junctional space. Moreover, it has become increasingly clear that for example glioma tumors traditionally targeted by Ad5 serotype adenoviruses often express only low levels of *CAR* and instead much higher levels of CD46 [43,44].

**Figure 2:**
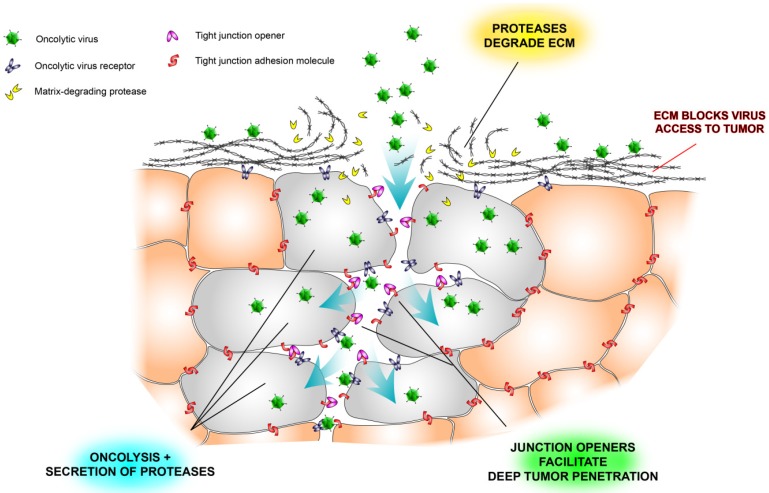
Schematic illustration of some physical barriers to oncolytic viruses and ways to overcome them. Tumor nests are often surrounded by extracellular matrix (ECM), which prevents viruses from reaching the tumor cells (see also Figure 1). Another problem is that tight junctions hide virus receptors and limit diffusion of viruses into the tumor tissue. Newly developed tight junction openers [40,47,48] may facilitate virus infection by exposing the hidden receptors, and virus-encoded proteases may degrade the stromal shield surrounding tumor nests [26].

Beyond that tight junctions hide multiple virus receptors, they contain a network of adhesion molecules, including *ZO-1*, cadherins, claudins and occludin, which, if perturbed, is associated with more aggressive disease in many types of cancer [45,46]. Since tight junctions also contain important receptors that mediate tumor-promoting signaling and which have been targeted by monoclonal antibodies, notably *Her2*, it would be highly useful to develop strategies that temporarily loosen the tight junction contacts—even at cost of a transient increase in tumor metastatic risk. One of the most interesting approaches has been to exploit the natural propensity of adenovirus serotype 3, which as part of its natural life cycle creates dodecahedral particles (PtDd) consisting of viral capsid proteins, penton base and fiber that open tight junctions by binding to and dissolving desmoglein-2 dimers and reducing E-cadherin expression, to develop a specific tight-junction opening molecule, JO-1 [40,47,48]. Adenovirus type 3 uses PtDds to promote its own infection, opening the tight junctions ahead of infection to maximize access to desmoglein-2. Analogously, when administered to human tumor xenografts, JO-1 facilitated penetration of trastuzumab much deeper into the tumor tissue than when the monoclonal antibody was administered on its own [47]. Moreover, JO-1 synergized with several chemotherapeutics in solid tumor models [48]. Backed by our own findings with oncolytic SFV, showing that both extracellular matrix and tumor cell compactness restrict virus spread and oncolytic efficacy, we believe combination approaches that target both tight junctions and extracellular matrix will prove effective in future virotherapy development (Figure 2).

An alternative approach to increasing tumor penetration by oncolytic viruses is to alter tumor cell morphology and status. Notably, cell death and in particular the type of cell death induced by virus has been shown to affect virus distribution within a tumor mass; in a study by Nagano *et al*., administration of apoptosis-inducing paclitaxel before injecting oncolytic herpes simplex virus increased virus dissemination in the tumor, allowing its diffuse in “tunnels” created by shrinking/dying tumor cells [49].

Maintenance of physiological adherence is essential for proper ECM function and for retaining cellular integrity. Therefore, a potential caveat of using ECM degrading proteolytic enzymes or tight-junction openers is the risk of neoplastic cell detachment from the tumor ECM and increased risk of metastasis. While ECM-degradation or tight junction opening may operate innocuously, there is also a chance that loss of E-cadherins via proteases or via non-specific deregulation of tight junction integrity during ECM-modulating therapy could trigger pro-tumorigenic Wnt/β-catenin signaling, possibly driving epithelial-to-mesenchymal transition [50]. One study showed that ectopic relaxin expression stimulated MMP expression and enhanced breast cancer invasiveness [51], whereas another paper found short term relaxin exposure to increase breast cancer cell motility whereas long term expression reduced both motility and cancer invasiveness [52]. In the context of oncolytic viruses, Lavilla-Alonso tested several proteases, including hyaluronidase, relaxin, and macrophage metalloelastase, and showed they all could assist adenovirus entry into tumors, yet, the authors did not detect treatment-induced metastases or increase in tumor invasiveness [53]. Another strategy possibly balancing some of the putative risks of tight-junction disruption could be to use Wnt-dependent oncolytic viruses [54].

### 2.4. Stromal Cells Hinder Viruses

Tumors are invariably fenestrated by host fibroblasts, myeloid cells and other non-transformed cells, which driven by cancer-induced cues can adopt various reprogrammed phenotypes to promote tumor angiogenesis and growth and to alter tumor responses to therapies [1,55]. As most of them normally play a role in immune homeostasis and pathogen sensing, they also respond to and influence oncolytic virus infection of tumors.

Fulci *et al*. reported that injection of oncolytic HSV-1 directly into glioblastoma tumor parenchyma triggered upregulation of CD68 and CD163 monocytic markers and rapid clearance of virus, likely executed by infiltrating phagocytic cells [56]. When clodronate liposomes were used to deplete macrophages *in vivo*, the authors observed a 5-fold increase in virus titers in the brain tumors concomitant with an 80% reduction in peripheral CD163^+^ macrophages in animal spleens, suggesting that CD163^+^ cells migrate into the tumors upon virus injection and limit overall oncolytic efficacy. While CD68^+^ cells were not reduced by peripheral macrophage depletion, arguing that these cells had been recruited to the tumors before the treatment, they could be eliminated in live glioma slices *ex vivo*, resulting in a 10-fold increase in virus replication. Macrophages may secrete antiviral type I interferon constitutively at very low (subnanomolar) levels [57] and recently Liu *et al*. showed that tumor-resident CD68^+^ macrophages induced a protective antiviral state in ovarian and breast tumors which led to resistance to oncolytic vesicular stomatitis virus (VSV) infection [58]. Type I *IFN* signaling (JAK1/2) inhibitor Ruxolitinib reversed the resistance, facilitating VSV replication in the macrophage-protected cancer cells. It is tempting to speculate that tumor-resident macrophages also rendered syngeneic GL261 and DBT mouse gliomas completely refractory to VA7 oncolytic alphavirus, as these cells were quite infectable *in vitro* and live glioma slices regained their sensitivity to virus within 24 h, indicating dilution of *IFN*-I [59,60].

Macrophages or the closely related myeloid suppressor cells are the predominant stromal cell type in many tumors and are actively recruited during cancer progression [61,62]. Heavy macrophage presence in the tumors indicates that such tumors may be resistant to oncolytic virotherapy, unless depleting preconditioning would be used. Moreover, as part of the early immune response to virus infection, monocytic cells are rapidly recruited to tumors following intratumoral virus injection. In one study, depletion of either CD11b^+^ cells or VEGF secreted by these cells in mice treated with oncolytic HSV-1 greatly increased oncolytic efficacy, tentatively linking pro-tumorigenic and antiviral angiogenic mechanisms [63].

Another key pro-tumorigenic signaling employed by many tumors is up-regulation of CXCR4, the receptor for chemokine CXCL12 (also known as SDF-1), and recruitment of stromal cells expressing CXCL12 which maintain the tumor and recruit additional cells to support tumor expansion and invasion, such as endothelial cell progenitors which contribute to neovascularization [64]. Indeed, high CXCR4 expression is a negative prognostic factor for many cancers. Oncolytic vaccinia virus engineered to express a soluble antagonist of CXCR4 showed improved intratumoral virus replication, increased vascular disruption and a markedly reduced infiltration of the tumors by putative immune-suppressive and tumor promoting stromal cells [65]. This study highlights how reducing pro-tumor stromal cell effects may alleviate antiviral resistance and yield increased overall therapeutic efficacy. On the other hand, if the paracrine antiviral effects of tumor-homing monocytic cells could be selectively abrogated these cells could favorably be used to ferry oncolytic viruses into tumors. Indeed, a significant delay in metastatic tumor potential was achieved when macrophages were harnessed to deliver a tumor-specific oncolytic adenovirus [66].

## 3. Tumor Cellular Defenses against Viruses

Improved oncolytic efficacy by means of increased tumor transduction is dependent on getting the viruses into the tumors and then on how many tumor cells are infected. Additionally, host immune status and the kinetics of the ensuing antiviral immune response are critical determinants of therapeutic efficacy with OVs. In order to improve virus infection of tumor cells and/or to limit infection of normal off-target cells, viruses may themselves be modified for increased receptor binding or uptake. Such strategies, summarized in greater detail elsewhere [67], include incorporation of receptor-binding motifs on the virion spikes, diverting infection to desired receptor-expressing (cancer) cells. Another strategy is to ferry the viruses in on the surface of or inside various secondary carrier entities, such as stem- or immune cells, which may enter the tumor from the circulation while paradoxically shielding the viruses from immune detection or antibody neutralization [68]. While these strategies ultimately increase the amount of virus that reaches the tumor bed, they are unlikely to alter tumor intracellular permissiveness to virus, which is still a prerequisite for oncolysis and likely primary determinant of virus-induced anti-tumor immune responses. In other words, tumor cells must allow viruses to replicate, otherwise there is no oncolysis and no therapy.

Central to cancer resistance to oncolytic viruses is the capacity to mount antiviral defense, and the quality of that defense. All human viruses have co-evolved with their hosts to achieve productive co-existence, and while innate antiviral defenses protect normal tissue from excessive virus replication, with the signaling and mechanisms described in great detail elsewhere [69,70], in tumors such defenses are an undesirable element that may restrict therapeutic efficacy. Propagating the defense are soluble cytokines, with type I (alpha and beta species) interferons playing an essential role against most viruses and type II (gamma) interferon providing a non-redundant auxiliary protective role in controlling pathogenesis of certain viruses. Mice knocked out for the type I *IFN* receptor (*IFNAR*) typically succumb within a day of multi-organ infection when challenged with viruses that may not even be pathogenic in normal adult hosts [71,72]. There have been no reported cases of genetic defects in the type I *IFN* receptor in humans, but three unrelated cases of complete signal transducer and activator of T cells (STAT) 1, the essential signaling mediator of type I *IFN*s, deficiency in humans have been recorded to date, all of which were lethal due to multi-organ virus infection [73,74]. While genetic defects in either type I or type II *IFN* genes are extremely rare in humans, genetic mutation of the *IFN* gamma receptor has been documented on some occasions, with the patients displaying high sensitivity to mycobacterial infections [75].

Some interferon-like proteins, such as limitin [76], and many unrelated and structurally diverse “danger”-associated endogenous molecules, including HMGB1 and heat-shock proteins, collectively called alarmins [77], likely signal via the type I *IFN* receptor or induce its expression, and therefore, in the coming chapters we consider tumor defense against viruses as an equation of the degree of type I *IFN* responsiveness. For tumors to be sufficiently infected by OVs to reach “reasonable” efficacy, some defects in tumor antiviral defenses are a prerequisite. Nevertheless, oncolytic viruses exert their efficacy not only by destructive replication in tumor cells but also by stimulating anti-tumor immune responses, and therefore overall efficacy of oncolytic viruses may be difficult to gauge based solely on capacity to replicate in cancer cells.

### 3.1. Innate Antiviral Defenses in Tumor Cells

What is the antiviral status in human cancer? Recent analyses from different normal cells that occupy the same organ reveal striking functional variation in components of the type I *IFN* signaling pathway that correlate with the tissue tropism and virulence of some virus strains [78,79]. Can similar variation in such components be found in cancer cells, or do cancer cells make an exception?

Transformed cells undergo selective elimination by the immune system before becoming cancerous, termed immunoediting, based on observations of differential capacity of immunocompetent mice to reject tumors previously grown in immunocompromised hosts versus syngeneic animals [80]. Cancer cell responsiveness to type I and type II *IFN*s plays a role during immunoediting: cancer cells that eventually progress to form a tumor display reduced capacity to respond to *IFN* gamma, which otherwise would upregulate MHC class I molecules and render the tumors amenable to CD8^+^ T cell-mediated destruction, whereas responsiveness to type I interferon initially helps tumors avoid immune purging during the editing phase and is then irrelevant [81]. Of note, the anti-tumor effects of type I *IFN*, still exploited today in several cancers, were shown to depend on host NK cells, and mice in which *IFNAR* was blocked by monoclonal antibody failed to reject even highly immunogenic tumors, as opposed to mice treated with control antibody, who all rejected the immunogenic tumor challenge [82]. Thus, whereas tumor responsiveness to type I *IFN* is not predetermined after immunoediting, other factors post immunoediting may influence it.

In several tumor types, varying levels of expression of components of the type I *IFN* signaling cascade have been detected, and in some tumor types these components have been found to have prognostic value, whereas in others they do not seem to influence survival [83,84]. However, emerging data suggests that antiviral genes may predict poor survival because they increase treatment resistance. In one study, poor prognosis of several types of cancer due to genotoxic treatment resistance was found to correlate with increased activity of *IFN*-I-pathway genes [85]. In this study, a triad of type I *IFN*-signaling pathway genes, *STAT1*, *ISG15*, and *IFIT1*, formed a pan-tumor-type negative prognostic factor, and a broader seven-gene cluster established negative prognostic criteria for treatment-resistant breast cancer. Treatment-resistance was subsequently shown to depend on STAT1 signaling; STAT1-expressing tumor clones were positively selected in animals *in vivo*, demonstrating increased proliferation and metastatic potential, and STAT1-expression mediated resistance against genotoxic assault by doxorubicin or ionizing radiation (knockdown of *STAT1* resulted in lower proliferation rate and metastatic capacity and increased sensitivity to genotoxic stress) [86,87]. In another study, resistance to epigenetic DNA modifiers 5-AZA-dC, a methyltransferase inhibitor, and LBH589 or MGCD0103, both histone deacetylase (HDAC) inhibitors, was correlated with increased expression of *IFN*-I-pathway genes in small lung cancer cells [88]. Basal *IFN*-related gene expression in several different SML cell lines was upregulated.

Interestingly, in many cancers, STAT proteins are not phosphorylated and persist in unphosphorylated form (U-STAT). Like its phosphorylated counterpart, U-STAT-1 increases expression of genes contributing to resistance to DNA damaging agents [89]. While it is currently unknown why cancers may prefer to use unphosphorylated forms, it is known that U-STATs activate distinct signals compared to their phosphorylated counterparts. However, importantly for oncolytic viruses, in many tumors, constant expression and release of type I *IFN* leads to a constitutively high level of unphosphorylated forms of *STAT1*, *STAT2* and *IRF9*, which together form an unphosphorylated but functional ISGF3-like complex that relocalizes to the nucleus and drives expression of a specific set of ISGs distinct from acute *IFN* I exposure [58]. These ISGs are sufficient to maintain a functional antiviral defense and they play a critical role in resistance to DNA damaging agents.

On a cellular level, virus replication is controlled by antiviral defense molecules [70], which in turn are primarily controlled by type I *IFN* signaling. In addition to classical type I *IFN*- or cytokine-induced antiviral signaling, tumors may employ other antiviral defense mechanisms. For example, stromal cells may secrete peptides with antiviral properties called defensins [90,91]. The defensins are amphiphatic and typically 29–42 amino acids in length and interfere with viruses by physically binding to and disrupting virus particles and/or by inducing antiviral responses in target cells via pattern recognition receptors. At least oral squamous cell carcinomas were found to overexpress defensins compared to normal control tissues [91]. While the role of defensins in oncolytic virus infection of tumors is unclear, one study showed that the immune-stimulating properties of defensins may be exploited to increase overall therapeutic efficacy. In this study, expression of beta 2 defensin from a conditionally replicating adenovirus yielded a superior therapeutic entity compared to the unarmed parental virus via enhanced anti-tumor immune responses mediated by *TLR4*-dependent activation of dendritic cells [92].

In summary, human cancers display varying degrees of expression of antiviral proteins or their signaling regulators at baseline. Some of these may increase in response to genotoxic anti-cancer treatments. In the next chapter we will highlight some of the consequences to oncolytic virotherapy of the heterogeneity in type I *IFN* responsiveness in tumor cells.

### 3.2. Oncolytic Virus Restriction by Innate Defenses

All oncolytic viruses tested to date display variable infectivity in different cancer cell lines. This variability is likely at least partly dependent on type I *IFN* as most oncolytic viruses are *IFN*-sensitive. *IFN*-secretion follows virus sensing by the cells via Toll-like receptors expressed on both the plasma membrane and in endocytotic vesicles, NOD-like receptors, *STING*, *AIM2*, *NLP* inflammosome and other danger sensing molecules, summarized elsewhere [93,94]. While all viruses carry an arsenal of counteracting molecules [69,95], no virus goes completely undetected. Even HSV amplicons, which are devoid of functional virus genes, induce a type I *IFN* response that mediates a STAT-1-dependent system-wide antiviral defense in mice within one hour of intravenous vector injection, resulting in significant reduction of transgene expression compared to expression in *STAT-1* knockout animals [96].

**Figure 3:**
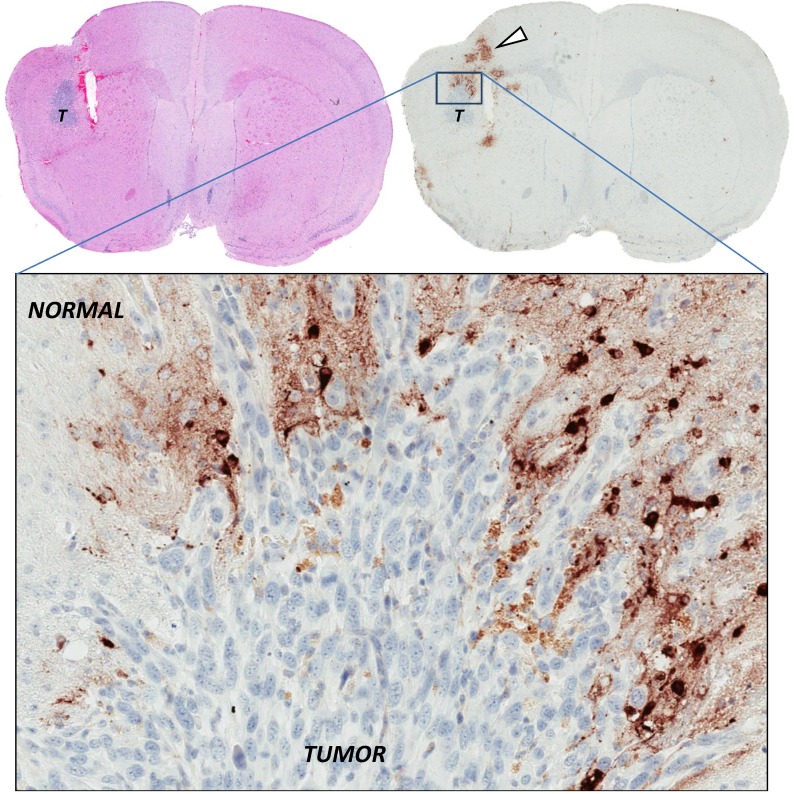
Restricted infection by oncolytic virus in the absence of physical barriers. Intracranial syngeneic Balb/c mouse DBT gliomas (T) were injected into the same stereotactic coordinates with high-dose oncolytic Semliki Forest virus (SFV) vector VA7. Brains were sampled and stained for SFV antigens 24 h post virus injection (in brown), showing that SFV predominantly infects normal brain parenchymal cells rather than glioma cells. DBT tumors are homogeneous and void of thick extracellular matrix deposits, as seen in Figure 1, arguing that tumor cells resist virus infection by other intracellular means [60].

During our own studies with attenuated SFV vector VA7 it became apparent that the dramatic and lasting therapeutic efficacy achieved following mere single intravenous (or intratumoral) injection of the virus in immunocompromised mice bearing human A2058 melanoma or U87 glioma xenografts [25,97] was not going to be recapitulated easily in immunocompetent animals [59,60,98]. The paradox in our studies was that while oncolytic SFV vector VA7 effectively replicated in and destroyed a variety of *IFN*-responsive cancer cell lines *in vitro*, it consistently failed to eradicate tumors generated from the same cell types *in vivo*, even if large doses of virus were injected directly into the tumor mass and even if the tumors were void of visible physical barriers (Figure 3). Conversely, only tumors generated from *IFN*-unresponsive cancer cells have seemed infectable *in vivo* by VA7 so far. In light of earlier findings by others, showing that type I *IFN* receptor knockout mice are highly susceptible to SFV infection and quickly succumb to multi-organ systemic infection [71], the parameters for oncolytic SFV efficacy seem clear: tumor cells must be defective in type I *IFN* response for the virus to be effective.

These findings are in line with emerging data from other groups, showing a remarkably strict dependence of oncolytic virus replication on defective type I *IFN* responsiveness, a dogma introduced at the turn of the 21st century [99]. For example, sarcomas and melanomas display differing permissiveness to oncolytic VSV, an obstacle which may be overcome by blocking type I *IFN* signaling [100,101]. In eight sarcoma cell lines, basal up-regulation of RIG-I and IFIT1 and rapid induction of *STAT1* phosphorylation upon *IFN* I treatment correlated with resistance to oncolytic measles virus [102]. In other studies, human pancreatic and ovarian cancer cells display resistance against Rb-dependent oncolytic adenovirus, strongly correlating with intracellular levels of *MxA*, and acquired resistance to repeated oncolytic adenovirus injections in an intraperitoneal ovarian carcinoma model was associated with an increase in MxA as well as several other key ISGs in the tumors [103,104]. A study comparing normal human or mouse melanocytes to a panel of melanoma cells revealed strong correlation between permissiveness to oncolytic VSV and capacity to mount antiviral defense in response to type I *IFN* [105]. In this study, VSV-rp30, harboring two mutations in *P* and *L* genes, was superior to several other tested oncolytic VSV strains, but the dependence on defective *IFN* responsiveness was still retained. In yet another study, oncolytic VSV replicated in four out of twelve mesothelioma cell lines which were unable to mount antiviral defense upon *IFN*-beta pretreatment or to respond to infection by up-regulation of *PKR*, *MxA* or *2'5'-OAS* mRNA [106]. In contrast, the non-permissive mesothelioma cells mounted antiviral defense, associated with *PKR*, *MxA* or *2'5'-OAS* mRNA up-regulation, in response to infection or to exogenous type I *IFN*. The authors of this study further linked the observed pattern of virus resistance to clinical mesothelioma samples, where in a mesothelioma tissue array, significant variance in immunoreactivity against PKR-, p48- and/or *IFN*AR was observed, with only a few samples displaying lack of reactivity against all three components, arguing that most tumors in clinical settings would display at least some level of resistance to oncolytic VSV.

Oncolytic adenoviruses are often deleted for E1A, which normally binds Rb and prevents it from sequestering E2F, which results in the viruses being unable to replicate in normal cells. However, since adenovirus, as with all viruses, contains elements that antagonize antiviral defenses, such elements have also been targets for deletion to render the virus sensitive to innate antiviral defenses (which would be expected to remain intact only in normal cells). Cancer cells expressing *Ras* may down-regulate protein kinase R and inhibit type I *IFN* induction by interfering with RIG-I signaling [107,108]. Also, select interferon stimulated genes appear to be repressed by the Ras/MEK/ERK signaling cascade [109]. *PKR* activation shuts down E2F-dependent translation, including translation of virus messenger RNAs. Therefore, an adenovirus deleted for VA RNA, which antagonizes *PKR*, should be capable of replicating in *Ras*-overexpressing (tumor) cells. However, while this was seemingly the case [110], it was later discovered that *Ras* is not an obligatory determinant for PKR-inactivation and that virus replication was halted even in *Ras* overexpressing cells via functional PKR-mediated E2F phosphorylation, implying that the parameters of VA RNA-deleted adenovirus replication had to be revised [111]. Similar findings were obtained with oncolytic reovirus, against which Ras-independent tumor resistance emerged *in vivo* [112]. Thus, under some circumstances even tumor cells over-expressing *Ras* may respond to type I *IFN* or to stimulants of pattern recognition receptors, activating *PKR* and inhibiting virus translation. To complicate matters, MEK-inhibitors have been shown restore antiviral signaling capacity in *Ras* overexpressing tumors [108,109]. This implies that human tumors treated with MEK-inhibitors, such as Trametinib which was recently approved for treatment of *BRAF*-mutated melanoma, may be poor targets for oncolytic viruses as antiviral signaling capacity in such tumors may be restored.

Some oncolytic viruses, such as members of the poxvirus family, are seemingly resistant to the antiviral effects of type I *IFN*s *in vitro* and may be controlled by other cellular factors [113,114,115,116]. However, in most cases, sensitivity or resistance to type I *IFN* is assessed *in vitro* and using only one cytokine, which may mask synergies with other factors present *in vivo*. One possible explanation for the *in vitro*–*in vivo* discrepancy is the tumor micro-environment, which not only imposes physical barriers to oncolytic viruses but also alters intracellular antiviral defenses. For example, glioma cells were found in the brain to spontaneously secrete type I *IFN*, which conferred resistance against oncolytic HSV. Resistance was linked to ECM protein *CCN1* interaction with glioma cell surface receptor alpha 6 beta 1 integrin, which activated interferon secretion [117]. It is tempting to speculate whether this or a similar ECM-glioma-interaction could have contributed to the resistance of syngeneic glioma cells we observed *in vivo* but not *in vitro* to both oncolytic SFV and vaccinia virus (FIG2; [60]). Also, while critical in many respects, antiviral signaling may occur without signaling via type I *IFN* receptor (*IFN*AR). For instance, upon infection of *IFN*AR KO mice with murine hepatitis virus (MHV), brain cells were found to have upregulated antiviral GTPase *TGTP* and *IFITm1* and *IFITm3*, all of which have confirmed antiviral activity *in vivo* against a variety of viruses [118]. In these cases, *IFN* gamma and *TNF* alpha likely account for at least part of the observed ISG activity, where e.g., *TGTP* is upregulated by *IFN*-γ signaling and *TNFα* has been shown to induce *ISG15* independently of *IFNAR* [119].

Interestingly, while most cancers are heterogeneous in antiviral defence signaling, one broad class of tumors in particular may display consistently low capacity to ward off oncolytic viruses; oncovirus-induced cancers. This is because oncoviruses, as all viruses, carry a complement of factors that abrogate antiviral sensors and effectors, which may result in tumors that are uniformly devoid of antiviral signaling. In an intriguing study, oncolytic VSV infected and destroyed human papilloma virus (HPV)-positive cervical carcinoma cells more effectively than HPV-negative head- and neck cancer cells due to HPV E6-mediated suppression of antiviral signaling [120]. HPV-positive or E6-expressing xenografts were efficiently eradicated from nude mice following VSV injection. The potential of this approach is that even oncolytic viruses that are extremely sensitive to type I *IFN*, such as attenuated SFV or M-mutated VSV, may function well against oncovirus-induced cancers where the oncovirus machinery ensures the lack of antiviral defense.

### 3.3. Exogenous Combination Therapy to Overcome Innate Defenses

While on first thought it would appear counterintuitive to antagonize the very factors that keep oncolytic viruses tumor-specific, emerging data shows that this can be accomplished in a safe way. One of the foremost strategies to increase oncolytic virus efficacy is to combine them with drugs that lower tumor antiviral defenses. In this regard, a primary target for interference is the prototypical type I *IFN* signaling cascade. While small molecular inhibitors against *IFNAR* are not available, activity of downstream signaling transducers may effectively be blocked. Human pancreatic cancer cells were shown to constitutively express high levels of MxA and OAS and to mount type I *IFN*-dependent resistance to oncolytic VSV. Resistance could be overcome by blocking activity of *IFN*AR-associated Janus kinase (*JAK*) 1 [100,113]. Similarly, *JAK*1/2 inhibitor ruxolitinib overcame type I *IFN*-dependent resistance of human SCC25 head and neck cancer cells against oncolytic VSV [121]. Ruxolitinib also successfully abrogated type I *IFN*-mediated antiviral effects elicited by macrophages [58], highlighting that this drug might also alleviate type I *IFN*-dependent tumor resistance to oncolytic viruses imposed by stromal cells. However, the obvious downside to using *JAK*1-inhibitors is their effects on critical host defense mechanisms, which may result in increased infection of normal cells. Moreover, JAK1-inhibitors may reduce anti-tumor immune responses elicited by oncolytic virotherapy. For example, ruxolitinib was shown to impair the capacity of dendritic cells promote CD8 T cell responses against model tumor antigens [122]. On the other hand, in this study clearance of adenovirus was delayed in ruxolitinib-treated normal animals, which makes it difficult to predict the overall impact of JAK1-inhibitors on tumor therapy where prolonged virus presence might translate to better therapeutic efficacy. Also, while the immune-stimulating and anti-tumor effects of type I and type II interferons may be adversely affected by *JAK*-inhibitors, central tumor-promoting cascades mediated by *JAK*s, such as *IL-6-STAT3*, may also be inhibited [123], yielding a difficult-to-predict net therapeutic outcome. At the moment, more studies are needed to establish the overall impact of interference with *JAK*1/2 signaling in cancer therapy.

Spurred by earlier reports of oncovirus reactivation and promoter enhancement by histone deacetylase (*HDAC*) inhibitors, this class of agents was assessed for capacity to enhance oncolytic virus potency by inhibiting antiviral responses. Indeed, such enhancement was observed with oncolytic VSV and SFV, and while it has been difficult to pinpoint the exact mechanisms that underlie *HDAC*-inhibitor enhancement of oncolytic virus replication in cancer cells, antiviral defenses in general are inhibited—for yet unknown reasons mainly in cancer cells and not in normal cells, which indirectly facilitates virus replication [124]. Interestingly, combination of *HDAC* inhibitors with conditionally replicating Δ24 E1A-deleted adenovirus gave improved virus replication rate and therapeutic efficacy in subcutaneous xenograft models only when the inhibitors were given before the virus, otherwise inhibition of replication was observed [125]. Similarly, *HDAC* inhibitors increased replication and oncolytic efficacy of HSV when given before the virus but not when given at the same time [126,127]. With nuclear DNA viruses, such as adenovirus and HSV, it is possible that *HDAC* inhibitors also alter virus genome accessibility to transcription factors, resulting in reduced replication or other adverse effects, implying that with these viruses *HDAC* inhibitors should be administered before the viruses.

Recently, it was found that *HDAC* inhibitor vorinostat activates *NfκB* signaling by facilitating hyperacetylation of the p65 subunit, which in turn activates cellular autophagy [128]. Autophagy was in another study shown to be important for VSV replication, presumably by inhibiting antiviral signaling. This is interesting, as a previous study revealed enhancement of VSV replication by inhibiting *TNF*-alpha-induced antiviral signaling mediated by p65 using NfκB inhibitors BMS-345541 or *TPCA-1* [129]. In this study, neither inhibitor had an effect on *STAT1* or *STAT2* phosphorylation or their nuclear relocalization in response to type I *IFN* but the authors observed clear induction of *ISG15*, *GBP1* and *MX1* in response to type I *IFN* in U87 glioma cells, with the latter two being dependent on *NfκB* signaling. Taken together, *NfκB* pathway activation may result in different antiviral responses in different cancer types, and inhibition or enhancement of oncolytic viruses by NfκB inhibitors is dependent on other concomitant cellular mechanisms, such as autophagy.

Since *HDAC* inhibitors modulate promoter activity/accessibility, it is conceivable other epigenetic or promoter modifying agents could work in a similar manner to repress antiviral defense induction and enhance oncolytic viruses [130]. DNA demethylating agents 5-azacytidine (5-Aza) and decitabine synergized with oncolytic herpes simplex type 1 virus in glioma models *in vitro* and *in vivo*, as did HDAC inhibitor valproic acid (VPA), which was already shown before to work well with oncolytic HSV as well as several other oncolytic viruses [131]. Moreover, inhibition of cancer antiviral defenses, specifically *RNAseL* and *PKR*, resulted when cancer cells were exposed to sunitinib, a multi-tyrosine kinase inhibitor originally developed as an inhibitor of *VEGF-R* and *PDGF-R* signaling, yielding strong anti-tumor synergy with *IFN*-sensitive oncolytic VSV [132]. Since sunitinib is approved for renal cell carcinoma and imatinib-resistant gastric cancers, combination with oncolytic VSV constitutes a promising and clinically relevant approach warranting further investigation. Interstingly, sunitinib as well as several other receptor tyrosine kinase inhibitors displayed antiviral effects against polyomavirus BK [133], arguing that these compounds are not universal virus enhancers.

Cyclophosphamide (CPA) is a general immunosuppressant used to minimize Treg presence during immunotherapy and also oncolytic virotherapy. CPA may, however, also enhance replication and efficacy of oncolytic viruses by diminishing cytokine secretion by stromal cells, including type I *IFN* [134]. Another compound enhancing oncolytic virus efficacy by lowering cell responsiveness to type I *IFN* is triptolide, which acts downstream of *IRF-3* activation [135]. Further, the kinase mammalian target of rapamycin (*mTOR*) enhances the antiviral effects of type I *IFN* by activation of its effector proteins 4E-BP, which binds and inactivates translation factor eIF4E, and S6K, which carries out cell-type specific signaling functions of type I *IFN*, such as activation of eukaryotic translation initiation factor 4B and promotion of *ISG15* transcription. Consequently, rapamycin was able to enhance oncolytic VSV replication and anti-tumor efficacy by inhibiting type I *IFN*-mediated antiviral signaling via *mTOR* [136].

Several other compounds synergizing with oncolytic viruses have been identified via high-throughput drug library screening, many of which seem to act by antagonizing antiviral defenses. For instance, a novel virus sensitizer, Vse1, was found to greatly diminish antiviral effects of type I *IFN* against oncolytic VSV in several cancer cell types, and the drug also synergized with the virus in subcutaneous mouse tumor models [137]. For many of these new compounds, the mechanism of action is still poorly understood, necessitating extensive safety studies to exclude undesirable effects against normal cells.

Apart from classical type I *IFN*-induced antiviral defenses, which typically prevent virus translation and degrade virus genomes, other cellular machineries regulate oncolytic virus efficacy. Notably, strategies to increase cancer cell death in response to oncolytic viruses have been tested. In a recent study, a class of compounds called SMAC mimetics synergized in several cancer models with oncolytic VSV by removing cancer cell block to apoptosis in response to virus-induced type I *IFN*, *TNF-α* or *TRAIL* [138]. Interestingly, since virus infection-triggered cytokines act on nearby non-infected cells, SMAC mimetics may in combination with oncolytic viruses also cause significant bystander tumor-destruction. Normal cells are not affected by the *IFN*-SMAC mimetic synergy, providing an important safety aspect. In another study, blocking the ER stress response circuitry triggered by oncolytic rhabdovirus infection by a small molecular inhibitor of serine/threonine protein kinase and endoribonuclease *IRE1α*, which was identified in a genome-wide siRNA screen, greatly enhanced cytotoxicity via caspase-2-induced apoptosis and increased oncolytic efficacy in refractory tumor models in mice [139]. This effect was independent on induction or responsiveness to type I *IFN*. Conversely, inhibitors of nucleoside transporter-1 (*ENT1*) were discovered from a high-throughput screen of enhancers of oncolytic HSV, and to date such inhibitors have not been reported to alter cellular antiviral responses [140]. Other compounds, such as common chemotherapeutics, and their mechanisms synergizing with oncolytic viruses have been discussed elsewhere—for most of them the possible role of antiviral defense antagonism in enhancing oncolytic virus efficacy remains to be studied [141].

Thus, many compounds are available to interfere with tumor antiviral defenses. The outstanding question for most of them, however, seems to be why such compounds do not render normal cells sensitive to oncolytic viruses. Elucidation of the exact mechanisms of action and the differences between normal and cancer cells constitute a worthy goal for studies in the near future.

### 3.4. Virus Engineering and Combination to Overcome Innate Defenses

Already in the 1950s it became clear that using certain pathogenic wildtype viruses, such as West Nile virus and Bunyamwera virus, in cancer patients would result in off-target toxicity [142,143]. Characteristic for pathogenic viruses is a greater ability to circumvent or antagonize cellular innate antiviral defenses than attenuated strains. For example, unlike the prototypical oncolytic reovirus strain type 3 Dearing, the T1L strain of reovirus causes accumulation of *IRF9* in the nucleus and inhibits activation of a select group of ISGs—yet because of this property the T1L strain is myocarditic unlike the oncolytic type 3 Dearing strain [78]. For these reasons, many oncolytic viruses used today, such as reovirus type 3 Dearing, oncolytic strains of Newcastle disease virus and M-mutated VSV have either a stronger capacity to induce or a weaker capacity to resist antiviral type I *IFN* than the corresponding wildtype strains [78,144,145].

An open question, however, is whether strain-specific or virus-specific differences that relate to specific components of the cellular antiviral machinery may be exploited for greater anti-tumor efficacy, *i.e.*, if one virus fails, could another be used in its stead? While Ras expression was critical for oncolytic reovirus replication in cells overexpressing *CUG2* oncogene, oncolytic vesicular stomatitis virus was unable to replicate in the same cells due to increased activation of *STAT1* and *OASL2* [146,147]. Thus, in these cells reovirus was not affected by *STAT1* or *OASL2*, and it therefore remains interesting to see which other antiviral effectors may have been lacking in the *CUG2-*expressing cells and, conversely, how reovirus and VSV differ in terms of sensitivity to antiviral effectors. While several oncolytic viruses have been compared to each other in terms of replication rate and cytotoxicity, systematic studies with regard to tumor antiviral defenses have not been conducted.

As any attenuated virus is likely to replicate effectively only in its own niche (*i.e.*, to be restricted by a specific set of ISGs), and as wild type strains may be too toxic for use in humans, a compromise may be achieved by incorporating select elements from wild type viruses into oncolytic virus backbones. Indeed, several chimeric and recombinant viruses have been generated that harbor specific elements of wild type strains or even of other unrelated viruses in order to better resist cancer antiviral defenses (Table 1). As an example of introducing wild type elements into attenuated viruses, Haralambieva reported on a measles virus Edmonston vaccine strain in which *P* gene was replaced with the counterpart from the wild type IC-B strain [148]. Later, Meng *et al*. included *N*, *P*, and *L* genes from wild type measles in Edmonston backbone, producing an enhanced but still safe oncolytic virus [149]. Edmonston vaccine virus induces a more robust *IFN*-response compared to wild type measles virus partly due to the weaker ability of V protein to suppress *MDA5*-mediated activation of IRF-3 and *IFN*-I induction [150,151]. *In vivo*, *P*-gene encoded proteins, particularly V, control the quality of the cytokine response to measles so that vaccine strains qualitatively induce a stronger inflammatory response compared to WT strains (where WT V protein suppresses multiple cytokine secretion). V protein short cytoplasmic tail has been shown to bind and inactivate *STAT2*, *IRF7*, *MDA5* and the Rel homology domain of the *NfκB* subunit p65 but not of p50 [152]. Also, V causes *LGP2* protein to bind and inactivate *RIG-I* [153], but it seemingly does not interfere with *TICAM-1* (*TRIF*, multiple *TLR* adaptor, including *TLR3*)-mediated *IFN*-β induction. Thus, if cancer and normal cells would differ with regard to *TICAM-1* or *IRF-3*, then such a virus could potentially target specifically cancer cells rather than normal cells. So far, the measles Edmonston-WT chimeras have proven safe in animals.

**Table 1:** Chimeric and recombinant *IFN*-I-antagonistic viruses. Example chimeric and recombinant viruses with oncolytic potential designed to overcome antiviral defenses. Not all constructs have been tested as oncolytics. Also, note that the nomenclature of vaccinia virus soluble type I *IFN* scavenger is ambiguous in the literature; “*B18R*” and “*B19R*” are often used interchangeably. Here we use *B19R* to indicate the type I *IFN* scavenger, which is the official term for the molecule from the Western Reserve strain.

Target virus	Target virus modifications	Donor virus	Donor gene(s)	Description	Reference
measles vaccine strain (Edmonston)	ΔP	measles wild type (IC-B)	*P*	wild type *P* gene product *V* is a stronger inhibitor of *MDA5*-mediated activation of IRF-3 and *IFN*-I than V from Edmonston strain	[148]
measles vaccine strain (Edmonston)	ΔN, P, L	measles wild type (IC-B)	*N*, *P*, *L*	compared to the construct above, addition of *N* and *L* created a chimera with stronger *IFN* antagonistic capacity	[149]
newcastle disease virus F3aa (lentogenic Hitchner B1)	F mutations conferring increased fusogenic capacity	Influenza A/Puerto Rico/8/1934 (PR8), H1N1	*NS1* (between *P* and *M*)	chimera showed superior oncolytic efficacy to parental virus	[154]
newcastle disease virus (mesogenic Beaudette C)		Influenza H5N1 or H1N1/09	*NS1* (between *P* and *M*)	while not tested as oncolytic viruses, these recombinants did display pathogenicity in chickens and increased capacity to replicate in human cells compared to parental virus	[155]
vaccinia virus (Western reserve)	ΔE3L	Influenza	*NS1*	This chimera has not yet been evaluated as an oncolytic agent. Vaccinia virus *E3L* is critical for replication in most cell types and for spread in normal mice by blocking *ISG15*—influenza NS1 partially restores the capacity to replicate in cells but the resulting chimera is still unable to spread in normal tissues *in vivo*	[156]
herpes simplex type 1 (Synco-2D)	γ34.5^−/−^, multiple mutations, expressing GALV under UL38 promoter	vaccinia virus	*B19R*	Vaccinia virus soluble type I *IFN* scavenger B19R facilitated replication and spread of oncolytic HSV. Oncolytic efficacy in animal models was increased	[157]
herpes simplex type 1	γ34.5^−/−^	human cytomegalovirus	*TRS1* or *IRS1*	*PKR*-antagonists *TRS1* and *IRS1* conferred increased replication capacity to oncolytic HSV-1, yielding greater therapeutic efficacy in glioma models in mice	[158]
vesicular stomatitis virus	ΔΜ51	vaccinia virus (Western reserve)	*B19R*	Superior ability to spread due to neutralization of paracrine type I *IFN*	[159]
maraba virus (MG1)	G protein (Q242R) and M protein (L123W) mutations	vaccinia virus (Western reserve)	*B19R*	Similar to the VSV recombinant but with the enhanced oncolytic capacity of the Maraba backbone. Virus was safe in mice	[159]

As an example of heterologous virus constructs, lentogenic Newcastle disease virus engineered to express influenza virus PR8 (H1N1) *NS1* showed enhanced oncolytic efficacy *in vivo* due to dampening of antiviral responses [154]. While such a virus did not display enhanced pathogenicity in that study, a mesogenic NDV virus harboring *NS1* from influenza strain H1N1/09 did show increased pathogenicity in chickens as well as an increased ability to replicate in human cells [155], raising some cautionary warnings for future engineering of similar replicative viruses. As another example, vaccinia virus soluble type I *IFN* scavenger *B19R* (NOTE: *B18R* is often used in the literature) has been engineered into both oncolytic HSV and oncolytic rhabdoviruses, resulting in all cases in more effective therapy agents without loss of tumor specificity [157,159]. Oncolytic *γ34.5* gene deleted HSV was complemented by two different *PKR*-antagonists from human cytomegalovirus, *TRS1* and *IRS1*, generating a chimera capable of reaching wild type virus replication levels in cancer cells *in vitro* and in tumor models *in vivo* [158].

The selection of various viral *IFN*-I-antagonists that potentially could be used to augment attenuated oncolytic viruses is vast [69,95]. For example, increased replication of several viruses, including HIV-1, Sindbis virus and E1-deleted adenovirus in mammalian cells was observed when the cells were engineered to express vaccinia virus E3L protein, influenza A virus *NS1* protein, ebola virus VP35 protein or HIV-1 Tat protein [160]. The choice of antagonist must primarily be made with safety in mind, but virus-specific and cell-specific differences in antagonist functions may potentially be exploited rationally when considering the overall combination. For example, vaccinia virus *E3L* is able to rescue VSV but not EMCV from exogenous *IFN*, whereas vaccinia virus *K3L* partially rescued EMCV but not VSV [161].

In anticipation of systematic, perhaps bioinformatically guided, “super-chimera” studies, two or more viruses that may complement each other’s shortcomings in antiviral defense antagonism have been considered. Notably, vaccinia virus was favorably combined with *IFN*-I-sensitive oncolytic VSV and SFV, yielding tumor-model-dependent increases in overall therapy efficacy that were dependent on vaccinia virus antagonism of type I *IFN* responses, which increased the replication of the *IFN*-sensitive viruses [37,60]. Such heterologous virotherapy approaches may also be used to generate immunological synergy, similar to heterologous prime-boost vaccination; anti-tumor immune responses may be increased due to targeting of the tumor by two different viruses (on two separate occasions), but anti-virus immune responses would be generated against different viruses each time, avoiding the problem of neutralizing immunity to and predominance of antivirus immune responses [162]. Some other ideas explored experimentally include engineering oncolytic viruses to express other oncolytic viruses. For example, the entire oncolytic parvovirus H-1 genome was placed under a regulatable promoter in oncolytic adenovirus, resulting in a more effective therapeutic entity than either virus alone without loss of tumor-specificity [163]. The genetic material of Semliki Forest virus replicons has been engineered into adenovirus and vaccinia virus backbones [164,165,166] and several other virus chimeras have been constructed [167]. However, the effects on tumor antiviral defenses of such a constructs remain to be studied.

## 4. Conclusions

The attribute “oncolytic” implies for a virus that it infects and, indeed, lyses the infected tumor. This property is primarily tested *in vitro* in cultured tumor cells. We have learned, however, that in the native tumor microenvironment in living hosts many viruses are no longer able to infect tumor cells or to kill them even if they manage to infect them. Tightly packed tumor cells and the network of supportive molecules of the extracellular matrix form a physical barrier to virus particle diffusion. It has also become evident that the tumor cells themselves may be much more capable of thwarting oncolytic virus advances than previously thought, with some tumor cells residing in a seemingly permanent non-permissive antiviral state. Tumors harbor multiple cell types in addition to the neoplastic cells, which may promote and propagate both physical and cellular virus resistance. As the efficacy of virotherapy in human cancer patients still falls shy of the achievements in animal models, it appears quite plausible that one or more of the barriers described in this review indeed constitute a real and formidable obstacle for oncolytic virus advancement into routine clinical use. Fortunately, some of the most difficult barriers have been identified, and a rapidly expanding arsenal of countermeasures is at our disposal. Our task is now to separate the wheat from the chaff and to systematically evaluate the proposed combination regimens that will yield the best results without compromising patient safety.
